# Impact of Body Mass Index on the Outcomes of Cryoballoon Pulmonary Vein Isolation for Paroxysmal Atrial Fibrillation

**DOI:** 10.3390/clinpract14060192

**Published:** 2024-11-12

**Authors:** Konstantinos A. Papathanasiou, Dimitrios A. Vrachatis, Charalampos Kossyvakis, Sotiria G. Giotaki, Gerasimos Deftereos, Maria Kousta, Ioannis Anagnostopoulos, Dimitrios Avramides, George Giannopoulos, Vaia Lambadiari, Gerasimos Siasos, Spyridon Deftereos

**Affiliations:** 1 2nd Department of Cardiology, National and Kapodistrian University of Athens, 11527 Athens, Greece; 2 Department of Cardiology, “G. Gennimatas” General Hospital of Athens, 11527 Athens, Greece; 3 3rd Department of Cardiology, Aristotle University of Thessaloniki, 54124 Thessaloniki, Greece; 4 Second Department of Internal Medicine, National and Kapodistrian University of Athens, Medical School, Attikon University Hospital, 12462 Athens, Greece; 5 3rd Department of Cardiology, National and Kapodistrian University of Athens, Medical School, Sotiria Chest Disease Hospital, 11527 Athens, Greece

**Keywords:** atrial fibrillation, obesity, body mass index, pulmonary vein isolation, cryoablation

## Abstract

Background: Atrial fibrillation (AF) is prevalent among obese patients, and cryoballoon ablation (CBA) is an effective strategy for the rhythm control of AF. The impact of body mass index (BMI) on the clinical outcomes of CBA for AF is not fully explored. Methods: 85 consecutive patients with paroxysmal AF were enrolled and were categorized into three groups as per their BMI: normal weight (BMI 18.5–25 kg/m^2^), overweight (BMI 25–30 kg/m^2^), and obese patients (BMI > 30 kg/m^2^). The primary study endpoint was a late (12 month) recurrence of AF. Early recurrence of AF, symptom improvement, and procedural outcomes were some key secondary outcomes. Results: 20 patients had normal weight, 35 were overweight, and 30 were obese. Obese patients featured a higher prevalence of diabetes mellitus, heavier exposure to smoking, and worse baseline symptoms (as assessed through EHRA class at admission and 12 months before CBA) compared to overweight and normal weight patients. Both late and early (<3 months) AF recurrence rates were comparable across the three groups. Of note, obese patients showed greater improvement in their symptoms post-CBA, defined as improvement by at least one EHRA class, compared to normal weight patients; this might be explained by improved diastolic function. Total procedure time and dose area product were significantly increased in obese patients. The multivariate logistic regression analysis indicated that early AF recurrence and the duration of hypertension are independent predictors of late AF recurrence. Conclusion: CBA is effective in overweight and obese patients with paroxysmal AF. Procedure time and radiation exposure are increased in obese patients undergoing CBA.

## 1. Introduction

Atrial fibrillation (AF) is the most prevalent heart rhythm disturbance, and it is estimated to affect more than 14 million people in Europe by 2050 [1,2]. Mendelian randomization studies have shown that obesity is causally associated with AF development [3,4], and it is known that increasing body mass index (BMI) confers an increased risk of AF development [5,6]. Furthermore, obesity often coexists with traditional cardiovascular risk factors that have been independently associated with the development of AF [7]. Of note, there is a bidirectional association between heart failure with preserved ejection fraction (HFpEF) and AF, the latter being an independent predictor of incident HFpEF [8]. Obesity is a principal factor in the development of both HFpEF and AF, yet recent advances in pharmacotherapy have shown promising results [9].

Randomized studies have previously suggested that weight reduction is beneficial for AF-related outcomes, such as arrhythmia free survival post-ablation [10], sinus rhythm maintenance [11], and AF regression [12]. Data from AF ablation registries have revealed that approximately 30% of the paroxysmal AF (PAF) patients undergoing ablation are obese (BMI > 30 kg/m^2^), highlighting the clinical relevance of obesity in view of ablation outcomes [13]. Furthermore, data from large European registries have indicated that obese patients undergoing radiofrequency ablation (RFA) for AF have higher arrhythmia recurrence rates on the long term [14,15].

Cryoballoon ablation (CBA) for AF is characterized by less procedural complexity, and it is non-inferior to RFA [16,17]. EARLY-AF trial evaluated CBA as a primary treatment for rhythm control in PAF patients and suggested that CBA-treated patients had lower AF recurrence rates and fewer hospitalizations during a follow-up period of three years [18]. In the coming years, the application of CBA for AF is bound to increase (especially in patients with prevalent comorbidities such diabetes mellitus and heart failure) [19,20], and its efficacy and safety in the treatment of overweight and obese patients with AF mandates further research.

The purpose of our study was to explore the impact of BMI on clinical and procedural outcomes of CBA for PAF patients. 

## 2. Materials and Methods

### 2.1. Study Design

#### 2.1.1. Population

Our study was a prospective, single-center cohort that enrolled consecutive patients undergoing cryoballoon pulmonary vein isolation (PVI) as a first ablation for PAF. Inclusion criteria were at least two symptomatic AF episodes within the last year and the failure of at least one class of antiarrhythmic drugs to prevent AF episodes. Patients who opted for ablation instead of antiarrhythmic medications were also eligible. Patients were excluded if they had non-paroxysmal AF, redo ablation procedure, left atrial (LA) diameter >50 mm, and a previous history of primary electrical heart disease (e.g., Brugada syndrome) or structural heart disease (e.g., prosthetic valve at any position, severe mitral stenosis or regurgitation, hypertrophic cardiomyopathy). Three groups of patients were created according to their BMI values: normal weight (BMI 18.5–25 kg/m^2^), overweight (BMI 25–30 kg/m^2^), and obese (BMI > 30 kg/m^2^). The study was carried out in accordance with the declaration of Helsinki and was approved by the responsible regional ethics panel (189/16-04-2020). All participants provided their informed consent. 

#### 2.1.2. Definitions and Investigations

Hypertension was defined as blood pressure ≥140/90 mmHg. Dyslipidemia was defined as total cholesterol >200 mg/dL or LDL-C > 130 mg/dL. Diabetes mellitus was defined as fasting plasma glucose >125 mg/dL or HbA1c ≥ 6.5%. BMI was computed as weight (in kilograms) divided by standing height (in square meters), and waist circumference was measured midway between the lowest rib and the iliac crest. All subjects underwent an echocardiographic study (iE33 machine, Philips Medical Systems, Andover, MA, USA) prior to ablation, and the ejection fraction of the left ventricle was calculated using the biplane method of discs (modified Simpson’s rule). Planimetered LA area in apical 4-chamber and apical 2-chamber views were utilized for the calculation of Left Atrial Volume index (LAVi). Echocardiographic data six to twelve months after the procedure were utilized to assess left ventricular diastolic function [21] and LA reverse remodeling [22,23,24].

### 2.2. Procedure

The CBA procedure was accomplished with a 28-mm cryoballoon (Arctic Front Advance Cardiac CryoAblation Catheter; Medtronic, Minneapolis, MN, USA). The ablation catheter was inserted into the LA following transseptal puncture, via a 12 French FlexCath steerable sheath, which was constantly flushed with heparinized saline. Electrical activity from each pulmonary vein was recorded with an octapolar, circular mapping catheter (Achieve, Medtronic). Right-sided pulmonary veins were isolated during the continuous pacing of the right phrenic nerve with a quadripolar electrode catheter placed in the superior vena cava. Antiarrhythmic medications were allowed over the 3-month blanking period post-ablation and were then discontinued, based on the discretion of the treating physician. 

### 2.3. Endpoints

The primary study endpoint was the first clinical recurrence of AF [diagnosed by 12-lead electrocardiogram (ECG) or a single-lead ECG tracing of ≥30 s] over a follow-up period of 12 months. Secondary study outcomes were early arrhythmia recurrence (ERAF) within the first 3 months, improvement in symptoms (defined by EHRA class change), AF-related hospitalizations, and procedural parameters such procedure time, LA dwell time, fluoroscopy time, and exposure to ionizing radiation.

### 2.4. Follow-Up

Patients were followed for three pre-specified hospital visits after the procedure (at 1, 3, and 12 months) for the assessment of symptoms and the detection of any AF recurrence (ECG and 24 h ambulatory ECG recording). If patients developed symptoms suggestive of AF recurrence, they were instructed to undergo symptom-guided ECGs.

### 2.5. Statistical Analysis

The data were assessed for normality through the Kolmogorov–Smirnov test. Continuous variables were given as means ± standard deviation (SD) if they followed normal distribution or as medians and interquartile range (IQR) if non-normally distributed. Categorical variables were summarized as relative frequencies. 

Comparisons between three groups were made using one-way ANOVA for the variables that followed normal distribution. Kruskal–Wallis test was used for skewed continuous variables. Comparisons between two groups were made using χ^2^ or Fisher exact tests for categorical variables. Normally distributed continuous variables were assessed with Student’s *t* test, while skewed continuous variables were assessed with the Mann–Whitney U test.

Receiver Operating Characteristic (ROC) curve analysis with the corresponding Areas Under the Curve (AUC) and multivariate binary logistic regression analysis were carried out to identify predictors of AF recurrence. First, we examined baseline variables in several univariate analyses, and afterwards we incorporated both clinically meaningful parameters and those proven statistically important in univariate analysis into a stepwise multivariate logistic regression model. A *p*-value < 0.05 was considered statistically significant. The data were analyzed using IBM SPSS Statistics for Windows, version 28 (IBM Corp., Armonk, NY, USA).

## 3. Results

### 3.1. Patient Population

85 patients with PAF (40% female, mean age 60 ± 10 years) were enrolled. 20 patients were grouped as normal weight, 35 as overweight, and 30 as obese. Obese patients were more commonly affected by diabetes mellitus and used to smoke more frequently as compared to overweight and normal weight patients. Additionally, obese patients featured worse symptoms at baseline, as assessed through their EHRA class at admission and the reported worst EHRA class during the previous year. Expectedly, obese patients had larger waist circumference compared to overweight and normal weight patients. Median CHA_2_DS_2_-VAS_C_ score was 1 (interquartile range 1–2) and did not differ between groups. A detailed description of the baseline features for the three groups is provided in Table 1. 

### 3.2. Atrial Fibrillation Recurrence and Secondary Outcomes

**Figure 1:**
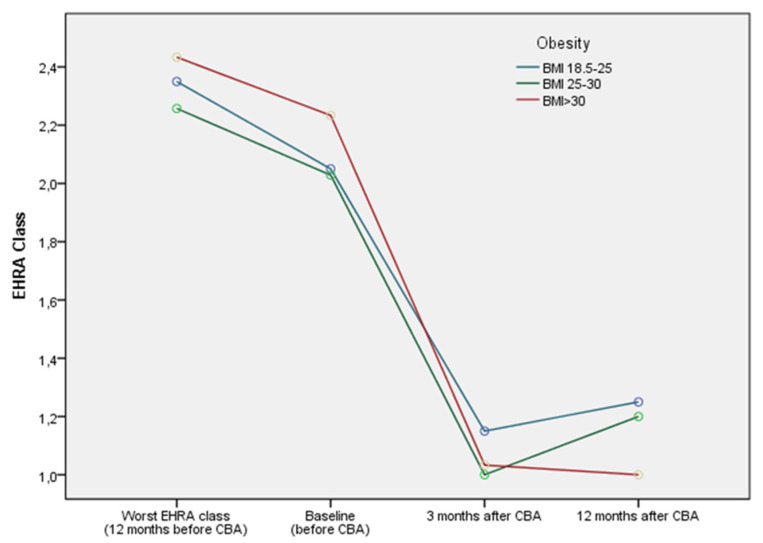
Estimated EHRA symptom class over the follow-up period for every group of patients separately. BMI: body mass index, CBA: cryoballoon ablation.

All patients completed 12 months of follow-up, and 75% remained free from arrhythmia. The late recurrence of AF did not differ across the three study groups (20%, 34.3% and 16.7%, *p*-value = 0.22, for normal weight, overweight and obese patients, respectively). In addition, ERAF rates were comparable among the three study groups, and overall, 8% of the patients had an AF-related hospitalization over the follow-up period. Of note, 88% of the patients reported the complete remission of their symptoms (EHRA class I) and obese patients featured greater improvement, since all of them had at least one EHRA class improvement in their symptoms as compared to 80% of the normal weight patients (*p* = 0.016) (Figure 1). 

In a post hoc analysis we found that the greater improvement in symptoms among obese patients might be at least partly explained by changes in echocardiographic indices of left ventricular diastolic function and LA reverse remodeling (defined as ≥15% reduction in left atrial volume after CBA). Grade of diastolic dysfunction, average E/e’, right ventricular systolic pressure, and LA reverse remodeling were statistically significantly improved only in obese patients over the follow-up period (Supplementary Table S1). In addition, a multivariate stepwise logistic regression analysis indicated that BMI is the only independent predictor of LA reverse remodeling (Supplementary Table S2). Finally, ROC analysis indicated that the diagnostic accuracy of BMI to predict LA reverse remodeling is modest (AUC: 0.66; 95% CI: 0.52–0.80; *p*-value: 0.04).

As far as procedural outcomes are concerned, total procedure time, radiation dose, and dose area product (DAP) were significantly increased in patients with BMI > 30 as compared to patients with BMI < 25. Fluoroscopy time and LA dwell time did not differ between the study groups. Of note, total procedure time was prolonged in patients with increased BMI (overweight and obese) due to more time needed to gain vascular access (Table 2). 

A sub-analysis comparing baseline features and study outcomes between patients without significant obesity (BMI < 30 kg/m^2^) and patients with obesity (BMI ≥ 30 kg/m^2^) indicated that obese patients are more likely to suffer from diabetes mellitus and have worse symptoms (as assessed through EHRA class) before the ablation procedure. Additionally, obese patients exhibited greater improvement in their symptoms after the procedure and a non-significant trend for decreased early and late arrhythmia recurrence rates compared to non-obese patients. Radiation exposure was significantly increased in obese patients (Supplementary Tables S3 and S4).

### 3.3. Predictors of AF Recurrence 

In the univariate logistic regression analysis, hypertension, duration of hypertension (years since the first diagnosis), and ERAF were associated with higher recurrence rates after CBA for the total population. Multivariate analysis revealed that both the duration of hypertension and ERAF could independently predict AF recurrence (Table 3). 

ROC analysis (the model included ERAF and duration of hypertension) suggested that the duration of hypertension features acceptable diagnostic accuracy in predicting AF recurrence post-CBA (AUC: 0.78; 95% CI: 0.63–0.92; *p*-value < 0.001) with a cut-off point of 8.5 years featuring 74% sensitivity and 89% specificity.

## 4. Discussion

The key findings of our study are that (1) CBA is clinically efficacious for patients with increased BMI, (2) radiation exposure is significantly increased in obese patients undergoing CBA, (3) improvement in symptoms post-CBA (as assessed through EHRA class changes) might be more pronounced in obese patients due to improved diastolic function, and, lastly, (4) duration of hypertension and ERAF are predictive of late AF recurrence post-CBA. 

Since obesity, HFpEF, and atrial fibrillation are interrelated, novel pharmacotherapies such as glucagon-like peptide 1 (GLP-1) receptor agonist might be proven a valuable addition in the therapeutic armamentarium for AF management. Recently published data suggested that GLP-1 receptor agonists lead to improvement in heart failure symptoms [9] and are associated with a reduction in AF recurrence post-ablation [25].

Only a few studies have explored the impact of BMI on CBA outcomes among AF patients. Weinmann et al. have studied a mixed population with paroxysmal or persistent AF undergoing CBA and found that obese patients are more likely to suffer from DM and become exposed to higher radiation doses as compared to normal weight patients. They also found that arrhythmia-free survival was comparable between BMI subgroups over a period of 48 months [26]. In a smaller study, CBA was found to be equally safe and effective for normal weight, overweight, and obese patients. Again, radiation exposure was significantly increased in obese patients [27].

Retrospective data from the Italian One Shot TO Pulmonary vein isolation (1STOP) registry confirmed that complications and arrhythmia recurrence rates post-CBA are comparable across patients with different BMI categories. In accordance with our findings, Malaspina et al. reported that CBA is associated with significant EHRA class improvement, regardless of BMI [28]. Scheurlen et al. found that obese patients (BMI > 35 kg/m^2^) are exposed to higher radiation doses and larger volumes of iodinated contrast media as compared to normal weight patients. They also compared RFA with CBA in obese patients undergoing PVI for AF and found that the former method is associated with reduced radiation exposure, yet a trend of increased late AF recurrence was noted (50% versus 24%, *p* = 0.09) [29].

All previous studies in the field have examined a mixed population of persistent and paroxysmal AF individuals undergoing first or redo ablation. We focused on patients with PAF receiving their first cryoballoon PVI and found that patients with increased BMI might experience greater improvement in their symptoms due to greater improvement in diastolic function indices and LA reverse remodeling compared to normal weight and overweight patients. Previous studies focusing on patients with AF and stable HFpEF suggest that PVI facilitates the regression of diastolic dysfunction [30], LA reverse remodeling [31], and improvement in peak exercise pulmonary capillary wedge pressure [32] and is associated with reduced AF readmissions [33] and all-cause mortality [34].

Although there are conflicting results regarding the impact of BMI on the duration of CBA procedure [35,36], the majority of the previously published studies indicated that increased BMI leads to significantly increased exposure to ionizing radiation during CBA [26,27,29,35,36,37]. Obese patients are at increased lifetime X-ray exposure risk due to prevalent comorbidities such as cancer, diabetes mellitus, coronary artery disease, and venous thromboembolism [38]. AF ablation-related exposure to ionizing radiation is not negligible, and CBA is associated with increased radiation exposure as compared to RFA [39]. Many patients with AF will ultimately undergo > 1 ablation procedure, and thus the cumulative exposure to ionizing radiation is further increased [40,41]. Data from the SWEET-Cryo cohort suggested that increasing BMI is associated with increased DAP (OR: 3; 95% CI: 1–8.3; *p* = 0.04) [42]. The optimal protection of patients and operators mandates a decrease in the use of fluoroscopy [43]. Although several contemporary studies have shown that various approaches, such as protocol adjustments, intracardiac echocardiography, and the KODEX system, are effective in reducing radiation exposure during CBA, most of them enrolled primarily non-obese patients [44,45,46].

The majority of the patients undergoing PVI for AF have class II or III EHRA symptoms [47], and the patients featuring a higher EHRA class are more likely to be hospitalized for AF [48]. We found that the vast majority of AF patients undergoing CBA are improved by at least one EHRA class over one year of follow-up, and our findings are in accordance with larger registries and randomized studies [49,50]. Hopefully, this improvement in symptoms might be maintained for at least four years [51]. We also observed a trend for fewer AF-related hospitalizations post-CBA in patients with increased BMI compared to patients with normal BMI, and that might be at least partly explained by the significant differences in the improvement of AF-related symptoms and left ventricular diastolic function between the groups. 

As far as late arrhythmia recurrence is concerned, we have previously shown that ERAF is an independent predictor of AF recurrence among CBA treated patients [52], and our current study is in accordance with this finding. In a recent and up-to-date meta-analysis, duration of AF, persistent AF subtype, ERAF, and LA diameter were predictive of AF recurrence three years post-CBA [53]. A novel finding in our study was the association between the duration of hypertension (years from the first documented diagnosis) and late AF recurrence. This might be explained by a more advanced atrial cardiomyopathic substrate in patients with a longer duration of hypertension, since we already know that both the duration and the stage of hypertension confer an increased risk of AF development, especially in patients with increased BMI or waist circumference [54].

The pathophysiology of obesity-mediated AF is complex, involving a multitude of neurohumoral, electroanatomic, metabolic, and inflammatory changes that lead to atrial cardiomyopathy. Obese patients with AF remain understudied in terms of both medical and interventional management [55]. Future studies should focus on better stratification [56] and individualized treatment strategies for overweight and obese patients [57].

### Study Limitations

This study is not without limitations. First, it was an observational study with a small number of participants, and thus no causal conclusions can be drawn. We would need a much larger sample size to detect small effect sizes of interest between BMI groups. The recruitment of such a population would be challenging in a low-volume center. Nevertheless, we explored whether BMI differences could show a trend in AF recurrence or secondary endpoints. Future studies with larger populations are needed to validate our results. Second, AF burden could not be assessed since our patients did not receive implantable loop recorders. Moreover, ECGs and 24 h ambulatory ECG recordings could have missed asymptomatic episodes of AF. However, we believe that no systematic bias was introduced because the same follow-up protocol was employed for all patients. Third, our findings are not applicable for patients with morbid or class II obesity. Additionally, LA volumes should ideally be assessed using a more precise volumetric method, such as the modified Simpson biplane method of discs. We also assessed patients’ quality of life only through changes in EHRA symptom class, and no AF-specific health-related quality of life questionnaire was utilized. Nevertheless, the validity of these questionnaires in everyday clinical practice remains questionable [58]. Lastly, the most recent guidelines for the management of AF suggest the use of CHA_2_DS_2_-VA score instead of CHA_2_DS_2_-VAS_C_, yet our study was conducted before this publication [7].

## 5. Conclusions

CBA is effective in overweight and obese patients undergoing first PVI for PAF. Obese patients might experience greater improvement in their symptoms due to improved diastolic function and are expectedly exposed to significantly higher radiation doses during CBA compared to non-obese counterparts.

## Figures and Tables

**Table 1:** Participants’ demographics.

	Total (N = 85)	BMI: 18.5–25 (N = 20)	BMI: 25–30 (Ν = 35)	BMI > 30 (N = 30)	*p*-Value
Age (years)	60 ± 10	62 ± 12	58 ± 10	61 ± 9	0.174
Gender (Female)	40%	45%	31.4%	46.7%	0.39
CAD	12.9%	14%	11.4%	13.3%	0.11
DM	17.6%	10%	11.4%	30%	**0.041**
HTN	52.9%	55%	42.9%	63.3%	0.23
Duration of HTN (years)	1 (0–9) ^±^	1 (0–10) ^±^	1 (0–9) ^±^	2 (0–6) ^±^	0.61
Smokers (active/ex)	22.4%/61%	15%/55%	28.6%/57%	20%/70%	0.48/0.46
Pack-years	7 (0–30) ^±^	1 (0–13) ^±^	10 (0–30) ^±^	15 (0–30) ^±^	**0.021**
Dyslipidemia	55.3%	60%	60%	46.7%	0.45
Body mass index (kg/m^2^)	28 ± 4	23 ± 1	28 ± 1.2	33 ± 2	**<0.001**
Waist Circumference (cm)	102 ± 9.6	90 ± 3	100 ± 4.6	112 ± 6.4	**<0.001**
Heart failure	3.5%	0%	2.9%	6.7%	0.48
Duration of PAF (years)	4 (2–10) *	5 (4–10) *	4 (1–10) *	3.5 (2–10) *	0.60
CHA_2_DS_2_-VAS_C_ score	1 (1–2) ^±^	2 (0–2)^±^	1 (1–2) ^±^	1.5(1–3) ^±^	0.35
HASBLED score	1 (0–2) ^±^	1 (0–2) ^±^	1 (0–2) ^±^	1 (0–1) ^±^	0.40
Anticoagulants	82.4%	75%	74.3%	96%	0.06
NOACs VKA	71.7% 10.6%	70% 5%	68.5% 5.7%	76% 20%	
Hypothyroidism	10.5%	8%	5.7%	16.7%	0.27
Depression	7%	4%	2.8%	13.3%	0.07
Family History of AF	18.8%	5%	28.6%	16.7%	0.06
PAD	4.7%	4%	5.7%	3.3%	0.53
Stroke	4.7%	4%	5.7%	3.3%	0.53
EHRA class (admission)	Class II: 89.4% Class III: 10.6%	95% 5%	97.1% 2.9%	76.7% 23.3%	**0.018**
Worst EHRA class within the last year (class III/IV)	32%	35%	20%	43.3%	**0.043**
LVEF (%)	59.5 (54–64) ^±^	59 (55–68) ^±^	60 (55–66) ^±^	59 (50–64) ^±^	0.45
LA diameter (mm)	41 ± 4.9	38 ± 5.5	40 ± 4.4	43 ± 4.5	**0.01**
LAVi (mL/m^2^)	31 ± 10	35 ± 15	29 ± 6	30 ± 11	0.42

AF: atrial fibrillation, BMI: body mass index, CAD: coronary artery disease, DM: diabetes mellitus, HTN: hypertension, LA: left atrium, LAVi: left atrial volume indexed, LVEF: left ventricular ejection fraction, Ν: number of patients, NOACs: non-vitamin K antagonist oral anticoagulants, PAD: peripheral arterial disease, PAF: paroxysmal AF, VKA: vitamin K antagonist. * median value and interquartile range, ^±^ = median value and minimum–maximum values.

**Table 2:** Primary and secondary study outcomes.

	Total (N = 85)	BMI:18.5–25 (N = 20)	BMI:25–30 (N = 35)	BMI > 30 (N = 30)	*p*-Value
LRAF (%)	24.7	20	34.3	16.7	0.22
ERAF (%)	9.4	10	14.3	3.3	0.31
AF admission (%)	8.3	5	11.8	6.7	0.63
EHRA class improvement (%)	89.4	80	85.7	100	**0.016**
Total procedure time (min)	92 ± 22	82 ± 20	94± 20	96 ± 23	**0.049**
Venous access time (min)	10 (6–15) *	5 (5–10) *	10(10–15) *	15(8–15) *	**0.015**
Time to transseptal (min)	16 ± 9	13 ± 7	16 ± 9	18 ± 9	0.16
LA dwell time (min)	60 ± 19	55 ± 13	60 ± 23	62 ± 17	0.40
Fluoroscopy time (min)	31 ±14	30 ± 18	32 ± 10	32 ± 13	0.25
Total radiation dose (mGy)	854 (522–1411) *	513 (459–563) *	879 (624–1302)	1289 (679–4041) *	**0.0001**
DAP (Gy*cm^2^)	71 (42–135) *	42 (34.8–49.6) *	81 (61–113) *	98 (52–150) *	**0.01**

BMI: body mass index. N: number of patients, LRAF: late arrhythmia recurrence, ERAF: early arrhythmia recurrence, LA: left atrium, DAP: dose area product. * Median value with interquartile range.

**Table 3:** Logistic regression analysis for late arrhythmia recurrence post-ablation.

	Univariate Analysis *p*-Value	Multivariate Analysis *p*-Value
HTN	**0.025**	0.11 (OR: 2.2; 95%; CI: 0.59–8.2)
Dyslipidemia	0.08	0.12 (OR: 6.2; 95%; CI: 0.69–55.3)
Heart failure	0.09	0.13 (OR: 6.6; 95%; CI: 0.57–77.2)
Duration of AF (years)	0.065	0.07 (OR: 1.1; 95%; CI: 0.88–1.39)
ERAF	**0.003**	**0.001** (OR: 27; 95%; CI: 4–195)
Duration of HTN (years)	**0.001**	**0.001** (OR: 1.4; 95% CI: 1.1–1.7)

AF: atrial fibrillation, CI: confidence interval, ERAF: early recurrence of AF, HTN: hypertension, OR: odds ratio.

## Data Availability

Data available upon reasonable request.

## Supplementary Materials

The following are available online at https://www.mdpi.com/article/10.3390/clinpract14060192/s1, Table S1: Left ventricular diastolic function and left atrial reverse remodeling over the follow-up period, Table S2: Predictors of left atrial reverse remodeling, Table S3: Baseline demographics according to obesity category, Table S4: Primary and secondary study outcomes according to obesity category.
