# Supplementary material for: Impact of Body Mass Index on the Outcomes of Cryoballoon Pulmonary Vein Isolation for Paroxysmal Atrial Fibrillation
Source: Clin Pract. 2024 Nov 12;14(6):2463–74. doi: 10.3390/clinpract14060192 (PMC11587088; doi:10.3390/clinpract14060192)
Supplement: Supplementary file 1 [file clinpract-14-00192-s001.zip › clinpract-3171902-supplementary.pdf]

Supplementary Table S1: Left ventricular diastolic function and left atrial reverse remodeling over the follow-up period.

	BMI: 18.5-25 (N=20)	BMI: 25-30 (N=35)	BMI >30 (N=30)	p-value*	p-value†	p-value‡	p-value§
**Diastolic dysfunction grade before procedure**				0.84			
**Grade 0-1**	60%	63%	60%				
**Grade 2-3**	40%	37%	40%				
**Diastolic dysfunction grade after procedure**				0.42	0.44	0.08	**0.02**
**Grade 0- 1**	65%	70%	77%				
**Grade 2-3**	35%	30%	23%				
**LA reverse remodeling**	5%	25.7%	30%	**0.04**			
**E/e' before**	9.8±4	10.5±3.7	10.5±4	0.12	0.63	0.13	**0.003**
**E/e' after**	10.6±3.5	9.6±3.1	8.4±3.1	0.88			
**RVSP before (mmHg)**	26±6	27±6	28±4	0.97	0.43	0.27	**0.01**
**RVSP after (mmHg)**	25±6	27±5	23±5	0.18			

BMI: body mass index, N: number of patients, LA: left atrial, RVSP: right ventricular systolic pressure, LA reverse remodeling: LA volume reduction ≥15% at 6 months

*comparison across BMI groups

† paired samples comparison for normal weight patients before and 6 months after ablation

‡ paired samples comparison for overweight patients before and 6 months after ablation

§ paired samples comparison for obese patients before and 6 months after ablation

Supplementary Table S2: Predictors of left atrial reverse remodeling.

	Odds ratio (95% Confidence Intervals)	p-value
**BMI (per kg/m^2)**	1.17 (1.02-1.33)	**0.02**
**Diabetes mellitus**	-	0.99
**Hypertension**	-	0.11
**Duration of hypertension (per year)**	-	0.88
**RAAS inhibitors**	-	0.99
**Age (per year)**	-	0.51
**Male gender**	-	0.39
**Early AF recurrence**	3.5 (0.95-13.3)	0.06

BMI: body mass index, RAAS: renin angiotensin aldosterone system, AF: atrial fibrillation

Supplementary Table S3: Baseline demographics according to obesity category

	Total (N=85)	Non Obese (N=55)	Obese (N=30)	p-value
Age (years)	60 ±10	59±11	61±9	0.38
Gender (Female)	40%	35%	46.7%	0.27
CAD	12.9%	12.7%	13.3%	0.45
DM	17.6%	11%	30%	**0.01**
HTN	52.9%	47.2%	63.3%	0.25
Duration of HTN (years)	1 (0-9)	1 (0-10)	2 (0-6)	0.77
Smokers (active/ ex)	22.4%/61%	23.6%/56%	20%/70%	0.43/0.65
Pack-years	7 (0-30)	4 (0-26)	15 (0-30)	0.54
Dyslipidemia	55.3%	62%	46.7%	0.12
Body mass index (kg/m^2)	28±4	26±2.2	33±2	**<0.001**
Waist Circumference(cm)	102±9.6	96±6	112±6.4	**<0.001**
Heart failure	3.5%	1.8%	6.7%	0.33
Duration of PAF (years)	4 (2-10)*	1 (5-10)*	3.5 (2-10)*	0.46
CHA_2DS_2-VASC	1 (1-2) ±	1 (1-2)±	1.5(1-3) ±	0.39
HASBLED score	1 (0-2) ±	1 (0-2) ±	1 (0-1) ±	0.49
Hypothyroidism	10.5%	7.3%	16.7%	0.25
Depression	7%	3.6%	13.3%	**0.01**
Family History of AF	18.8%	20%	16.7%	0.78
PAD	4.7%	5.4%	3.3%	0.35
Stroke	4.7%	5.4%	3.3%	0.56
EHRA class (admission)	ClassII:89.4%<br>ClassIII:10.6%	96% 4%	76.7% 23.3%	**0.02**
Worst EHRA class within last year (class III/IV)	32%	25%	43.3%	0.31
LVEF (%)	59.5 (54-64)±	60 (56-66)±	59 (50-64)±	0.07
LA diameter (mm)	41±4.9	39 ±4.6	43±4.5	**0.001**
LAVi (ml/m^2)	31±10	31±10	30±11	0.73

BMI: body mass index, N:number of patients, CAD: coronary artery disease, HTN: hypertension, DM: diabetes mellitus, PAD: peripheral arterial disease, AF: atrial fibrillation, PAF: paroxysmal AF, LVEF: left ventricular ejection fraction, LA: left atrium, LAVi: left atrial volume indexed.* median value and interquartile range, ±= median value and minimum-maximum values

Supplementary Table S4: Primary and secondary study outcomes according to obesity category.

	**Total (N=85)**	**Non-Obese (N=55)**	**Obese (N=30)**	**p-value**
LRAF (%)	24.7	29	16.7	0.08
ERAF (%)	9.4	11	3.3	0.18
AF admission (%)	8.3	9	6.7	0.69
EHRA class improvement (%)	89.4	83.6	100	**0.04**
Total procedure time (min)	92 ±22	87±20	96±23	**0.05**
Venous access time (min)	10 (6-15) *	10 (5-15) *	15(8-15) *	0.39
Time to transseptal (min)	16±9	15±9	18±9	0.22
LA dwell time (min)	60±19	57±19	62±17	0.18
Fluoroscopy time (min)	31 ±14	30±11	32±13	0.67
Total radiation dose (mGy)	854 (522-1411) *	624 (513-1034)*	1289 (679-4041)*	**0.02**
DAP (Gy*cm^2)	71 (42-135)*	62 (41-113)*	98 (52-150)*	**0.04**

BMI: body mass index. N:number of patients, LRAF: late arrhythmia recurrence, ERAF: early arrhythmia recurrence, LA: left atrium, DAP: dose area product

*Median value with interquartile range
